# Beyond cholesterol: linking the conformation of apolipoprotein B to atherogenesis

**DOI:** 10.1097/MOL.0000000000000997

**Published:** 2025-06-23

**Authors:** Katariina Öörni, Martina B. Lorey

**Affiliations:** a Molecular and Integrative Biosciences, Faculty of Biological and Environmental Sciences, University of Helsinki; b Wihuri Research Institute, Atherosclerosis Research Laboratory, Helsinki, Finland

**Keywords:** aggregation, apolipoprotein B-100 structure, atherogenicity, conformation, proteoglycan binding

## Abstract

**Purpose of review:**

This review integrates recent structural and biochemical insights into apolipoprotein B (apoB) containing lipoproteins to highlight how factors beyond cholesterol levels contribute to atherosclerosis.

**Recent findings:**

Emerging evidence demonstrates that the atherogenic potential of apoB-containing lipoproteins varies substantially both between and within lipoprotein classes. Recent studies using high-resolution cryo-electron microscopy, crosslinking mass spectrometry, and computational modeling reveal that even subtle differences in lipoprotein composition, particle size, and lipid spatial organization can significantly alter the conformation and dynamic behavior of apoB on the particle surface. These conformational shifts influence a variety of lipoprotein characteristics such as the stability of the particle, their ability to interact with receptors and enzymes, and their proatherogenic potential as measured by the propensity of lipoproteins to bind to proteoglycans of the arterial wall or to undergo modification and aggregation.

**Summary:**

In this review, we discuss how novel structural and functional information can refine our understanding of the distinct properties of apoB-containing lipoproteins and their role in atherosclerosis and lipid accumulation. Understanding of the specific features related to the proatherogenic behavior of the lipoproteins helps in understanding the complexities of atherogenesis and cardiovascular risk beyond cholesterol.

## INTRODUCTION

Cholesterol delivered into the arterial wall by LDLs and other apolipoprotein (apo) B-containing lipoproteins is a key driver in the initiation and progression of atherosclerosis [1,2]. The apoB-lipoprotein family is heterogeneous, encompassing chylomicrons and their remnants, VLDLs, IDLs, and LDL, each of which can be further divided into (multiple) subclasses [3]. Among these, LDL is the most abundant, accounting for up to 90% of apoB-containing particles [1]. With the exception of the largest particles (diameter > 70 nm), virtually all apoB lipoproteins are capable of crossing the endothelial barrier into the subendothelial intima, where their cholesterol cargo accumulates and sparks plaque formation. As such, both elevated particle numbers and the cholesterol they carry are recognized as major risk factors for cardiovascular disease.

Recent studies have revealed that the atherogenicity of apoB-containing lipoproteins varies substantially both between and within lipoprotein classes, giving rise to the concept of “relative per particle atherogenicity” [4,5]. This concept suggests that inherent differences in the proatherogenic properties of individual lipoproteins contribute to overall cardiovascular risk [3]. In parallel, the “cumulative exposure hypothesis” emphasizes that both the level and duration of exposure to elevated circulating lipids are critical determinants of atherosclerotic cardiovascular disease (ASCVD) risk [6]. Together, these concepts underscore the need for personalized risk determinations and prevention strategies. 

**Box 1:**
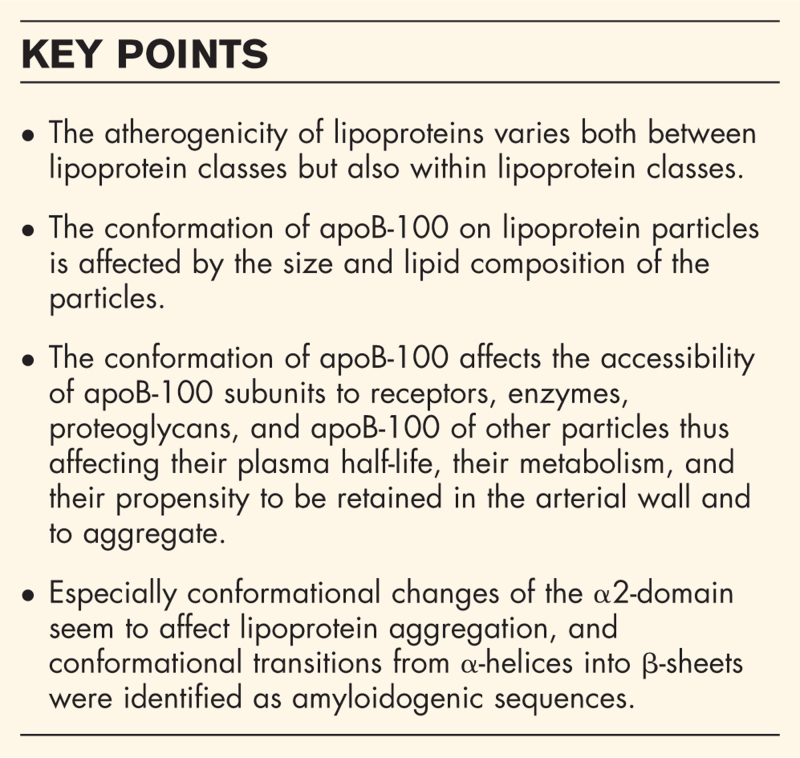
no caption available

**FIGURE 1:**
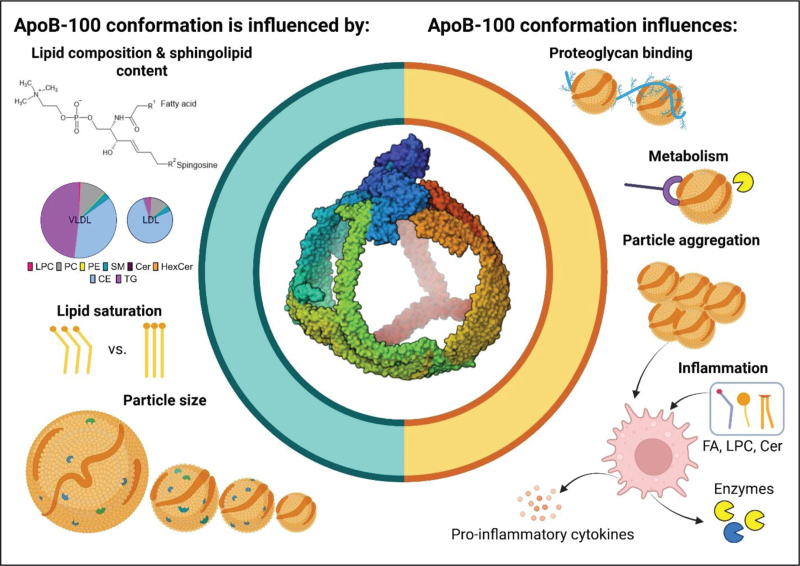
Apolipoprotein B-100 (https://doi.org/10.2210/pdb9EA7/pdb) is depicted on LDL based on data from [7^▪▪^]. Conformation of apoB-100 is influenced by the lipid composition, overall lipid saturation, and the lipoprotein size. The conformation, in turn, influences lipoprotein metabolism and proatherogenic properties of the lipoproteins. FA, fatty acid, LPC, lysophosphatidylcholine, Cer, ceramide. Created in BioRender.

Advances in structural biology through high-resolution cryo-electron microscopy (cryo-EM), cross-linking mass spectrometry, and integrative computational modeling have recently provided insights into the architecture of apoB-containing lipoproteins [7^▪▪^,^8^^▪▪^,9^▪^,10^▪^]. These studies suggest that the spatial organization of lipids within the lipoprotein particles profoundly influences the conformation and dynamics of apoB on the lipoprotein surface, thereby affecting particle stability, receptor interactions, and proatherogenic potential (Fig. 1). In this review, we discuss how these novel structural insights can refine our understanding of the distinct properties of apoB-containing lipoproteins and their role in atherosclerosis and lipid accumulation.

## STRUCTURAL HETEROGENEITY OF APOB-CONTAINING LIPOPROTEINS

ApoB-containing lipoproteins exhibit remarkable heterogeneity in both composition and size. They span from triacylglycerol-rich chylomicrons and VLDLs to cholesteryl ester-enriched LDL. Chylomicrons, derived from the intestine and containing the truncated version of apoB, apoB-48, can reach diameters of several hundred nanometers. In circulation, chylomicrons are lipolyzed by lipoprotein lipase, generating chylomicron remnants. VLDL particles are secreted by the liver and contain the full-length apoB-100. They are also triacylglycerol-rich, have diameters up to 90 nm, and their progressive lipolysis produces IDL and subsequently LDL. The smallest LDL particles have diameters of less than 20 nm.

Cholesterol within lipoproteins is present in two distinct forms. Unesterified cholesterol is primarily located in the surface monolayer alongside phospholipids and sphingolipids, whereas cholesteryl esters (CEs) reside in the hydrophobic core. The fatty acid composition of the CEs is determined in part by the substrate specificity of sterol O-acyltransferase 2 in intestinal cells and hepatocytes, but also by the available fatty acids derived, for example from the diet.

Advances in cryo-EM and complementary biophysical techniques have challenged the traditional view of apoB-containing lipoproteins as uniformly spherical particles. Thus, VLDL and IDL display polyhedral morphologies in cryo-EM [11,12]. This feature was suggested to facilitate lipid transfer processes mediated by cholesteryl ester transfer protein. LDL particles, on the other hand, typically appear in cryo-EM as flattened discs with a layered core of CEs interspersed with regions of varying density [9^▪^,^13^,^14^]. The different CE species have varying fatty acid chain lengths and unsaturation levels have been suggested to form specific domains of varying density in the lipoprotein core [9^▪^].

Combination of lipidomic profiling with NMR-based analysis demonstrated that differences in serum CE species correlate with distinct lipoprotein profiles and cardiometabolic risk factors [15]. For example, CE (16 : 1) is associated with higher risk, while CE (20 : 4) tends to associate with a cardioprotective risk profile. Although it remains to be determined whether these or other specific CE species are direct actors in atherosclerosis or simply markers of distinct metabolic pathways, our lipidomic analysis of ultracentrifugally isolated lipoproteins following a 28-day supplementation with 4 g/day of icosapent ethyl (fatty acid [20:5]) indicates distinct metabolic differences between incorporation of the fatty acid into CEs and other lipid species between VLDL, LDL, and HDL [16].

The advances in structural biology have also redefined the conformation of apoB-100 on lipoprotein particles. Integrative approaches combining cryo-EM, cross-linking mass spectrometry, high-speed atomic force microscopy, and state-of-the-art computational refinement have elucidated a highly dynamic architecture for apoB-100 [7^▪▪^,^8^^▪▪^,9^▪^,10^▪^]. The models depict apoB-100 as composed of a large, globular N-terminal domain that transitions into an extended amphipathic β-sheet “belt” wrapping around the lipid core. This belt includes multiple flexible loop regions that likely enable the protein to adapt its conformation in response to large shift in the particle size from large VLDLs to smallest LDLs. Interestingly, in apoB-100 on VLDL particles, the number of intermolecular interactions appeared to be smaller than in LDL particles and no long-distance cross-links were observed [7^▪▪^,10^▪^]. These findings were suggested to result from apoB-100 being more submerged within the lipid moiety of VLDL.

Familial hypercholesterolemia disrupts lipoprotein metabolism primarily through mutations in the LDL receptor or apoB-100, impairing the clearance of LDL particles and prolonging their time in circulation. This has the potential to lead to unique structural and compositional changes in LDL particles. Indeed, in a recent study, LDL from patients having familial hypercholesterolemia were found to be smaller and to have a higher and more ordered CE content than LDL from controls [17]. In addition, there were differences in the conformation of apoB-100. Such conformational changes may influence the interaction of LDL with LDL receptors. A recent cryo-EM study shed light into the intricate interaction of LDL particles with LDL receptor [8^▪▪^]. The interaction was even more complex than envisioned previously involving two receptors, each binding an LDL particle with several interactive sites.

## RETENTION OF APOB-CONTAINING LIPOPROTEINS IN THE ARTERIAL WALL

A fraction of the circulating apoB-containing lipoproteins will enter the arterial wall, where they can be retained. The largest particles do not traverse a functional endothelium, but most of chylomicron and VLDL remnants and all IDL and LDL particles can cross the endothelial cell layer [3]. In the arterial wall, the lipoproteins encounter a tight extracellular matrix network. Binding of apoB-containing particles particularly to the glycosaminoglycan (GAG) chains of proteoglycans is considered the initiating key event of atherosclerosis [18–20]. The various apoB-containing particles differ in their ability to bind to the proteoglycans, VLDL having the lowest and small LDL particles having the highest affinity [21]. A likely reason for the variation in particle affinity is the difference in the conformation of apoB-100: the recent studies suggest that apoB-100 in VLDL is less accessible that apoB-100 in LDL particles [7^▪▪^,10^▪^] (Fig. 1).

Binding of apoB-containing lipoproteins to proteoglycans is highly sensitive to the local extracellular pH [22]. In the early stages of atherosclerosis, the pH is generally neutral. However, as local inflammation develops, similarly to what is observed at other inflamed sites, the extracellular environment becomes more acidic [23]. This acidification enhances the ionic interactions between proteoglycans and apoB-containing lipoproteins [24]. Specifically, the negatively charged GAG chains on proteoglycans interact with positively charged regions of apoB. Histidine residues, with pKa of approximately 6.0, become increasingly protonated as the pH drops, thereby amplifying the positive charge of apoB and strengthening the interaction. Indeed, an ex-vivo study demonstrated that at acidic pH, blocking the positive charge on histidine residues significantly inhibits the binding of LDL to human coronary artery sections [25^▪^]. These findings suggest that local pH shifts can markedly enhance the retention of apoB-containing lipoproteins in the arterial wall.

In a recent review article [26], various possibilities to reduce lipoprotein retention as options to reduce atherosclerosis were discussed. The possibilities included small chemical inhibitors targeting GAG chain elongation, monoclonal antibodies against sulfated GAGs, or apoB-100 mimetic peptides that interfere with the binding of lipoproteins to the proteoglycans. Of these, prevention of GAG chain elongation by targeting the relevant signaling pathways with small chemical compounds was considered to have the most potential, as this approach could specifically reduce lipoproteins retention without affecting the physiological functions of proteoglycans. The affinity of lipoproteins to proteoglycans can also be used to study their potential to be retained in the arterial intima. Such analyses are already in use in research settings. For example, our laboratory uses a microtiter well based assay to measure the binding of lipoprotein-derived cholesterol to isolated human aortic proteoglycans. Using this assay, we have observed diet-induced differences in the proteoglycan-binding of lipoproteins [16,27,28].

Binding of LDL particles to heparin introduces irreversible conformational changes in apoB-100 that increase the affinity of LDL for heparin and promote LDL oxidation, proteolysis, and lipolysis, and reduce the stability of the particles [29]. If binding of LDL to proteoglycans induces similar changes, retention of LDL would promote their modification by local oxidants, proteases, and lipases. While heparin and arterial proteoglycans both contain the GAG component, heparin is characterized by a highly sulfated, linear polysaccharide chain, whereas the proteoglycans consist of a protein core with one or more covalently attached GAG chains that exhibit variable length, branching, and sulfation patterns. Therefore, it is not clear whether the binding of LDL to proteoglycans would also induce significant changes in the conformation of apoB-100. However, early studies using phospholipase A_2_ to modify LDL suggest that this would be the case [30,31].

## MODIFICATION OF APOB-CONTAINING LIPOPROTEINS IN THE ARTERIAL WALL

Retained apoB-containing lipoproteins are susceptible for oxidizing agents and enzymes, proteases, and lipases [32,33]. Indeed, lipoproteins isolated from atherosclerotic lesions show signs of multiple modifications and they are often aggregated and fused [34,35]. The progression of atherosclerosis is strongly driven by such modifications: First, the lipoproteins themselves undergo a series of biochemical alterations, and they become oxidized, proteolyzed, and lipolyzed. These processes promote aggregation and fusion of the lipoprotein particles and can lead to formation of cholesterol crystals. Lipoprotein aggregates have a more difficult time for leaving the arterial intima as they bind more tightly to components of the extracellular matrix [32]. Second, the products of these modifications, most notably the lipolytic products such as fatty acids, ceramides, and lysophospholipids, can activate local inflammatory cells and the endothelium and trigger inflammatory responses [36]. Third, the modified and aggregated lipoproteins are taken up by phagocytes and induce foam cell formation and a robust inflammatory response [37]. In addition, macrophages have the possibility to create “lysosomal synapses” into which they can secrete lysosomal enzymes to hydrolyze aggregated lipoproteins extracellularly [38].

In addition to LDL, also VLDL and IDL have been shown to aggregate upon modification [39]. Whether the accumulation of these triacylglycerol-rich lipoproteins in atherosclerotic lesions is a similar process as LDL accumulation is not yet known. One possibility is that triacylglycerols or their lipolytic products promote local inflammation or cause endothelial dysfunction and thus promote lipoprotein or inflammatory cell entry to the lesion [36]. However, triacylglycerols do not accumulate in the atherosclerotic lesion [40], and lipoprotein lipase that is secreted locally by macrophages has the potential to hydrolyze the triacylglycerols [41].

The susceptibility of LDL particles for aggregation varies from donor to donor and depends on the lipid composition of the particles: particles enriched in sphingolipids aggregate more readily than particles enriched in phosphatidylcholine [42]. Clinical relevance of the aggregation-propensity of the LDL particles has been shown in individuals having established coronary stenosis or peripheral artery disease [42,43]. In these studies individuals having aggregation-prone LDL were more likely to suffer a major adverse cardiovascular event than the other groups. Familial hypercholesterolemia patients, whether children or adults, are also characterized by having aggregation-prone LDL [17,44].

LDL aggregation has been shown to depend on conformational changes in apoB-100 [45] (Fig. 1). The α2-domain apoB-100 has been identified to contain sequences that, when misfolded, are particularly prone to aggregate, which can even lead to formation of amyloid-like structures [46]. In a recent computational study, specific amyloidogenic sequences that undergo conformational transitions from α-helices into β-sheets were identified in this part of apoB-100 [47]. Interestingly, we have previously observed that reduction in α-helical content of apoB-100 is a prerequisite of sphingomyelinase-induced LDL aggregation [45].

A recent study investigated the proteolytic landscape of human atherosclerotic plaques using N-terminal proteomics [48^▪^]. Among the findings, apoB was identified as a significant contributor to neo-N-terminal peptides, particularly in lipid-rich atherosclerotic plaques, suggesting enhanced proteolytic activity targeting apoB in the lipid-rich plaques. Interestingly, the cleavage sites of apoB peptides varied between plaque types, indicating distinct protease activity in different plaque environments. Secretion of the apoB peptides can occur during foam cell formation, but an interesting possibility is that the peptides are generated extracellularly. Secretion of lysosomal enzymes has been reported from activated macrophages [38,42].

Understanding of the aggregation process has led to studies examining various therapeutic strategies to prevent LDL aggregation [49]. Successful strategies have in common their ability to preserve the native conformation of apoB-100. For instance, apoA-I mimetic amphipathic peptide 4F integrates into the LDL surface phospholipids and stabilizes apoB-conformation and reduces LDL aggregation [50], while DP3, a retro-enantio peptide derived from LDL-receptor like protein 1, binds directly to apoB-100, maintaining its α-helical structure and reducing aggregation and atherosclerosis [51^▪^]. These approaches have demonstrated efficacy in reducing atherosclerotic plaque formation in experimental models, underscoring the potential of targeting apoB-100 conformational stability as a promising strategy.

## CONCLUSION

Recent cryo-EM studies have provided remarkable insights into LDL particle structure and the dynamic conformation of apoB-100. These analyses reveal that apoB-100 exhibits significant conformational versatility, yet a degree of structural stability is essential for maintaining particularly LDL integrity. Notably, the conformation of apoB-100 appears to be largely influenced by the lipid composition of the particle; even subtle variations in the lipid milieu can induce considerable changes in the protein's overall structure. On the other hand, differences in the conformation of apoB-100 influence lipoprotein metabolism as well as various proatherogenic processes including lipoprotein retention, modification, and aggregation. A deeper understanding of the intermolecular interactions within these lipoproteins may allow us to design strategies that stabilize lipoprotein particles. This could, in turn, enhance their binding to the LDL receptor promoting efficient clearance from circulation, or reduce their affinity for subendothelial proteoglycans, thereby diminishing arterial retention. Furthermore, in the case of modified lipoproteins, emerging evidence suggests that small peptides capable of stabilizing apoB-100 conformation might inhibit particle aggregation and ultimately reduce atherosclerosis.

## Acknowledgements

*Figure 1* *was created in BioRender.*

### Financial support and sponsorship

*The authors have received grants from the Finnish Foundation for Cardiovascular Research, Sigrid Jusélius Foundation, and the Finnish Cultural Foundation.*

### Conflicts of interest

*K.Ö. is an inventor in a patent on LDL aggregation.*

## REFERENCES AND RECOMMENDED READING

Papers of particular interest, published within the annual period of review, have been highlighted as:

▪ of special interest
▪▪ of outstanding interest
